# Surgical correction of the webbed neck: an alternative lateral approach

**DOI:** 10.3205/iprs000106

**Published:** 2017-03-02

**Authors:** Imen Mehri Turki

**Affiliations:** 1 Maxillo-Facial and Aesthetic Surgery Department, Mohamed Tahar Maamouri Teaching Hospital, Nabeul, Tunisia

**Keywords:** webbed neck deformity, Turner syndrome, surgical technique, pterygium colli

## Abstract

**Objective:** The webbed neck deformity or pterygium colli is the number one symptom of the Turner syndrome that leads the patient to consult a doctor. Various but rare surgical approaches have been described to correct this deformity. We reviewed our experience with the surgical correction of the pterygium colli.

**Methods:** Through five clinical cases, we describe the surgical technique with a lateral approach which provides a better control of the operative site, allows for the excision of the underlying trapezial fascial web, thus preventing recurrence seen in the posterior approach, and restores a normal hairline.

**Results:** No postoperative wound infection occurred. No recurrence was observed through 24 months. Three patients developed hypertrophic scars.

**Conclusion:** The lateral approach associated with an advanced flap and a Z-plasty is an effective technique for correction of this neck deformity. The presence of a multidisciplinary team, formed with maxillofacial and plastic surgeons, endocrinologists and psychologists, is required to treat these patients allowing reintegration into society and family.

## Introduction

Turner syndrome affects about 1/2500 female births worldwide. This syndrome is caused by complete or partial loss of one X chromosome. In some girls, a number of features are apparent such as: webbed neck or pterygium colli, low hairline at the back of the head, lymphedema of hands and feet, broad chest with widely spaced nipples, heart defects, kidney problems, ovarian dysgenesis, and poor growth [1].

Because of the aesthetic and psychological harm, these girls ask for surgical correction of the webbed neck at school age. Many techniques have been described to correct this deformity. We expose our surgical method with a literature review.

## Clinical cases

**Figure 1:**
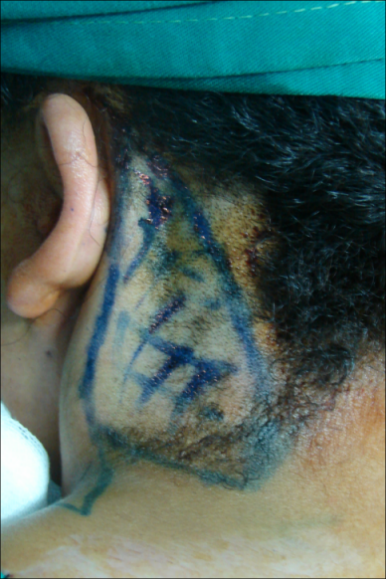
Preoperative drawing: the harmful triangular skin next to the fibrous band

**Figure 2:**
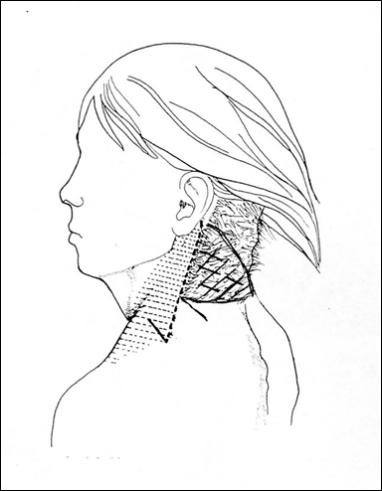
Our technical approach: lateral incision (dotted line) allows the dissection of the anterior cervical skin with the excision of the fascial band. The Z-plasty performs the basicervical shape. The triangle represents the surface of skin excision.

**Figure 3:**
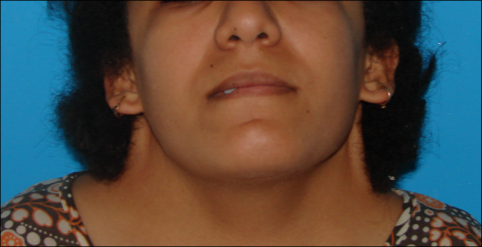
Preoperative photo

**Figure 4:**
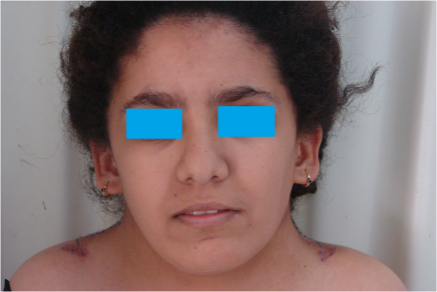
Photo one month after surgery

Five girls between 17 and 19 years old came for surgical correction of their pterygium colli with low and laterally displaced nuchal hairline. For all these girls, the surgical method was the same. They underwent it under general anesthesia. The girl was placed in a prone position for bilateral repair (Figure 1 (Fig. 1)). The incision was made at the junction of the hairless skin and the hairy skin from the mastoid until the lower ending of the webbing skin. An undermining of the subcutaneous skin was done on the antero-lateral direction exposing the fibrous fascial band which must be excised to prevent recurrence. Obtaining the hedged cutaneous surface, the harmful skin having a triangular shape was excised with respect to the future hairline. Skin closure was done after postero-superior translation of the lateral cervical flap associated to a single Z-plasty on the ending of the incision regarding to the acromion (Figure 2 (Fig. 2)). The follow-up was done with an average of 24 months. The hyperthrophic scars observed in three patients were treated by corticotherapy injection and silicone sheet application. No functional deficit was found and the range of motion of the head and shoulders was completely free in all these girls. In addition, the morphological appearance of the neck and the placement of the hairline were correct allowing these girls a better social integration (Figure 3 (Fig. 3), Figure 4 (Fig. 4)).

## Discussion

The first description of the webbed neck was reported by Kobylinski in 1883 [2] and the name “pterygium colli” was coined by Funke in 1902 [3]. It corresponds to bilateral dermal webbing that can be identified and rolled between the fingers. It extends from the mastoid to the acromion. The excess of the skin is associated with a thickening of the superficial cervical fascia sometimes entangled by fibers of the platysma muscle. The etiopathogeny of this deformity is discussed. Some authors attribute it to the spontaneous resolution of a cervical cystic hygroma before 16 weeks gestation. This hygroma is secondary to lymphatic malformation, particularly dilated jugular lymphatic sacs that result from a blockage in the venous drainage system. Von Kaisenberg revealed the hypoplasia of lymph vessels in the superficial dermis by using immunohistochemical markers [4].

Girls with Turner syndrome present a hairy posterior face of the pterygium colli continued by a low hairline next to the sixth and seventh cervical vertebrae. The aim of the surgical treatment is to create a normal neck contour with a symmetrical postero-lateral hairline by excising a triangular cutaneous band as done in our patients [5]. 

Chandler in 1937 was the first to describe the surgical correction of pterygium colli using Z-plasties. However, the skin of the anterior basicervical region becomes hairy by the transposition of the posterior skin of the pterygium colli [6].

Foucar (1948) described a posterior approach. Through a midline incision in T, two lateral flaps are detached and then translated into a posterior-superior direction to erase cervical webbing. The excess of scalp is excised [7]. To avoid visible scars, Shearin and Defranzo [8] used a posterior butterfly incision in which the loose skin and subcutaneous tissue was pulled to a midpoint on the back of the neck. The fibrous band was never excised and recurrences were usually seen [9]. Rather than the posterior approach, Menick in 1984 had the idea of using a lateral approach to create a cervical advancement flap and closure with a shape of two “Y”s, without associated Z-plasty [10]. 

Niranjian [11] and Miller [12] conducted skin expansion for patients respectively 16 and 30 years old. At this age, the biomechanical properties of skin differ from those in children.

S. Murthy and M. McGraw recently described a lateral approach called M to T rearrangement. By this technique, the trapezial fascial web is directly exposed and completely excised [13].

## Conclusion

We believe that the posterior approach doesn’t allow for complete excision of the trapezial fascial web giving more recurrence. In addition, this approach makes it difficult to control the anatomical components, particularly the external jugular vein, cervical plexus, VII and XI nerve, unlike the lateral approach. 

## Notes

### Competing interests

The author declares that he has no competing interests.
